# Seeking healthcare at their ‘right’ time; the iterative decision process for women with breast cancer

**DOI:** 10.1186/s12885-020-07520-x

**Published:** 2020-10-19

**Authors:** Anita Eseenam Agbeko, Joshua Arthur, Jonathan Bayuo, Basil Benduri Kaburi, Ishmael Kyei

**Affiliations:** 1 Department of Surgery, Komfo Anokye Teaching Hospital, Kumasi, Ghana; 2 Public Health Unit, Komfo Anokye Teaching Hospital, Kumasi, Ghana; 3 Department of Nursing, Faculty of Health and Medical Sciences, Presbyterian University College Agogo, Agogo, Ghana; 4 Ghana field epidemiology and Laboratory training Programme, School of Public Health, University of Ghana, Accra, Ghana

**Keywords:** Advanced breast cancer, Health seeking behaviour, Care seeking pathway, Delay

## Abstract

**Background:**

About 85% of breast cancer patients attending Komfo Anokye Teaching Hospital (KATH), Ghana, present with stage III/IV disease. In spite of great investments into the early diagnosis and management of breast cancer, late presentation persists and poses a barrier to realising the possible benefits of the gains made in breast cancer management. This study assessed the symptom appraisal and medical health seeking behaviour of women with either locally advanced or metastatic breast cancer attending breast clinic at KATH.

**Method:**

In-depth interviews of women presenting with clinical stage III/IV breast cancer were conducted to explore the women’s care seeking pathways after symptom identification until arrival at KATH from May 2015 to March 2016. Thematic data analysis was conducted using the Andersen behavioural model for health service use.

**Results:**

Fifteen women aged 24–79 years were interviewed. The time from symptom identification to reporting to KATH was 4–24 months. The initial symptom was a breast lump or breast swelling which all the women identified themselves. These were initially appraised as not serious because most importantly, they did not interfere with their daily function. Symptom progression such as prevented them from undertaking their usual economic, social and family function triggered seeking care from health facilities. The availability of money to pay for care and diagnostic investigations influenced the time taken to navigate the referral pathway. While the women initially deferred healthcare for reasons related to their ability to perform economic, family and social roles, ultimately, aggressively pursuing healthcare was also for the same economic, family and social reasons or goals.

**Conclusion:**

Deciding to seek care and pursue treatment for breast cancer symptoms may be much more complicated than it appears. Economic, family and social function significantly drive the health seeking process both at the personal and health facility phases of health seeking. Breast cancer education messages must be adapted to incorporate these functional goals and their influence on symptom appraisal and decision making to seek help and not just focus on the breast symptom as an isolated entity.

## Background

Breast cancer diagnosis at a late stage is a challenge in low and middle-income countries, with 30–98% of breast cancer cases diagnosed at stage III or IV [1]. In some African countries, 70–80% of breast cancer patients present late [2–4]. Of an estimated 1.38 million women diagnosed with breast cancer annually, almost 50% of cases and 58% of resulting deaths are from developing countries [5]. Among African women, advanced stage at diagnosis, aggressive tumour type, poor differentiation and triple negative hormone receptor status are contributing factors [1, 6, 7] to this poor phenomenon. Furthermore, initiating definitive treatment more than 3 months after patients’ discovery of symptoms contributes to the high mortality rates [8]. At the Komfo Anokye Teaching Hospital (KATH) in Ghana, about 85% of breast cancer patients present with stage III/IV disease [7].

Many women discover their breast symptoms themselves [9], mostly by chance [10], or during activities such as bathing, dressing, or breast feeding [11]. Subsequently, their interpretation of the symptoms is the first step of the health seeking process [12]. Women have delayed seeking care because they have initially interpreted their breast symptoms as not being serious [10, 12–17]. Some have attributed their breast symptoms to hormonal changes [16], trauma [18], or breastfeeding [12]. When breast symptoms have not met their expectations of breast cancer, it can be evaluated as not being serious. Women’s expectations of how breast cancer symptoms present vary. A painless pea-sized breast lump [13], or breast pain, have been considered to be cancer [16]. Some believe breast lumps turn into cancer if often pressed or touched [19]. Some studies suggest the absence of pain has been interpreted as an unserious breast cancer symptom [12, 16, 18]. Aside awareness of breast lump, poor knowledge about other early signs of breast cancer also contribute significantly to delayed health seeking [4, 20–23].

Besides symptom appraisal outcomes, poverty [4, 24–26], psychological factors such as fear and denial [12, 18, 23, 27, 28], and the use of complementary and alternate medicine [4, 22, 23, 26, 27, 29] have been identified as contributing to late health seeking behaviours. The impact of socio-demographic and economic factors such age, education, marital status, area of residence, and income level on breast cancer health seeking behaviour have been reported to be equivocal [4, 14, 30–36].

Symptom interpretation is the most important step in the health seeking process for cancer diagnosis and this is deemed to contribute to 60–80% of the health seeking process [37]. Women who delay seeking help continue to actively monitor their symptoms and do seek care when they perceive their symptoms to have become serious [12, 13, 16]. Eventually, there is always a trigger that drives them to seek help, albeit too late in most cases. Such triggers include the onset of pain [12, 16, 18, 38], increasing severity, and the persistence of the identified symptom such that it interferes with daily activities [18, 39]. What informs the behaviour of women who seek help only when they perceive worsening symptoms? What goes on during that period of active monitoring of their symptoms? There has been great investments into screening, early diagnosis, and management of breast cancer. However, late presentation poses a barrier to realising the possible benefits of advanced breast cancer screening and management modalities available today. It remains a healthcare challenge that some women still present to healthcare facilities with advanced breast cancer – localised or metastatic. This study assessed the symptom appraisal and medical health seeking behaviour of women with either locally advanced or metastatic breast cancer attending the breast clinic at KATH.

## Methods

### Study design and setting

We conducted a phenomenological study using a descriptive qualitative design among breast cancer patients accessing care at the breast clinic of the Komfo Anokye Teaching Hospital (KATH), Ghana, from May 2015 to March 2016. Komfo Anokye Teaching Hospital is a 1300-bed capacity tertiary hospital that provides surgical, chemotherapy, and radiotherapy treatment for breast cancer. The Hospital has a dedicated breast care centre where women with breast symptoms are first seen. All decisions on breast cancer treatment are taken by an interdisciplinary tumour board comprising surgeons, oncologists, pathologists, radiologists, nurses, and social workers.

### Participants, recruitment and sampling

We purposively recruited fifteen (15) women presenting for the first time to the breast clinic of KATH with breast disease clinically suggestive of Stage III or IV breast cancer as defined by the American Joint Committee on Cancer [40]. The clinical stage was determined by the attending physician based on history, physical examination, and prior laboratory or radiological investigations the women may have done. These women would have invariably observed overt changes in their breast during the period before seeking care at KATH breast clinic. Eligibility also included reporting to KATH breast clinic at least 3 months after identifying the symptom. Age was not a limitation to inclusion. Women who were too frail, such as those who were visibly tired out after the routine clinical consultation and examination were not approached for interviews. The clinic nurse approached eligible participants face to face and explained the study and its purpose to them using the participant information leaflet. Women who voluntarily expressed their interest to participate were directed to contact the researcher in person. Two eligible women declined participation, one due to how the interview time will affect her travel schedule and the other because she felt anonymity had been breached by the researcher referring to her by her name during the interview.

### Data collection

Each participant signed a written Informed consent prior to her interview. Each of the in-depth interviews was conducted in a private room in the breast clinic at KATH by AEA, who was a PhD candidate at the time. The interviews were guided by open ended questions to explore how the women noticed their breast symptoms; how the symptom had evolved since; the experiences that influenced their decision to seek medical care and the activities they had undertaken until arriving at the breast care center. In order to promote a dialogue that could explore their responses, the questions were used flexibly and the order adapted and elaborated to suit each interview context [41]. Data saturation was reached by the 14th interview and confirmed with 15th. The interviews, which were audio recorded, were conducted in the local dialect of Twi and translated into English during transcription. Each interview lasted for about 40–45 min. Back-translation was used reiteratively during the transcription and analysis process to ensure the meaning of the original responses was not lost [42].

### Data analysis

The data was coded and analysed using a deductive thematic approach with priori themes guided by the Andersen Behavioural Model of Health care utilisation [43]. The Andersen Behavioural Model describes the determinants of health service use to include individual characteristics such as demographic factors, cultural norms, education, occupation, social relationships, health beliefs, personal and family income/wealth, individual-perceived need for health care; and health service factors such as cost and accessibility to care, and patient’s satisfactions and perception of the care provided.

## Results

Fifteen (15) women aged between 24 and 79 years, were interviewed. Ten (10) of them had clinical stage III and five (5) had clinical stage IV breast cancer. Four (4) of the women lived in the Ashanti Region (i.e. same region as the Komfo Anokye Teaching Hospital) while the other eleven (11) lived outside the region. Travel to the clinic was typically by public transport and took anywhere from thirty (30) minutes to ninety (90) minutes for those within the same region, and from two (2) to nine (9) hours for those coming from outside the Ashanti Region.

All the participants except for one (Int. 2) lived with some family members, that is their husband, children, grandchildren, siblings, parents or other extended family members. One (1) participant was a retired civil servant who received a regular pension. Seven (7) of the participants were traders, and three (3) farmers, all of whom had an irregular income from their occupation. The remaining four (4) were unemployed and relied on family members for remittances.

The health seeking journey for the women unfolded in two phases: the personal phase and the health system/facility phase. These phases unfolded either separately or concurrently.

### The personal phase of health seeking

#### Symptom identification

All fifteen (15) women identified their breast change themselves except for one whose lump was noticed by her friend. They were provoked by breast campaign messages or a sense of discomfort/pain to perform self-breast examination (SBE). The first sign identified by most of the interviewees was a lump, whilst others first experienced a breast swelling, or breast pain as the index symptom.

*“I saw it myself that the breast looked a bit swollen” [Int. 11]*

*“There was something in it, something hard and they have been announcing that when there is something in your breast go and have it checked” [Int. 4]*

#### Personal beliefs and appraisal

Many of the women thought their first symptom was nothing serious. Their reasons included the small size of the lump that was unapparent to others, and the normal gross appearance of the breast. More importantly, ‘serious signs’ expected to be associated with serious illness such as pain or interruption of one’s normal activities, were absent.

*“It wasn’t big and I thought it won’t worry me” [Int. 5]*

*“It was hard but it was not paining me and I could wear a dress and go to the farm” [Int. 1]*

They appraised their breast symptom regularly, and several factors played a role in deciding what it was, and what action to take. One such factor was common cultural or traditional knowledge of breast illness, lumps, or body swellings and how they are commonly managed. Such swellings were commonly regarded as boils which were expected to discharge pus and subsequently resolve, sometimes with the application of locally prepared topical treatment. Previous experience of similar changes amongst family or friends strengthened this expectation.

*“The way it was hurting I was thinking, as for something that is swollen, it will burst and discharge then you are free and that’s all” [Int. 6]*

*“I had seen women with breast disease, some their breast became very huge* [and] *they used herbal medicine at home and for the majority it resolved” [Int. 10]*

#### Breast cancer campaign messages on symptom appraisal

Nine (9) of the women had been exposed to breast cancer messages via mass media (television, radio, posters) or local church/social groups. They understood that breast lumps could be cancers, and that prompt medical attention should be sought if breast lumps are found. They had also acquired skills to perform routine breast self-examination (BSE). However, they did not appear to know that other traditionally known breast symptoms could be cancers, nor how different breast swelling evolved over time. Additionally, they appeared not to know what other signs, aside breast lumps, were suggestive of breast cancers.

*“I hear always about breast diseases….as for the breast I did not really see anything in it. It was my armpit that I noticed something” [Int. 9]*

*“Our pastor’s wife is a nurse and taught us periodically to examine our breast. She said breast can develop cancer so you must take the right steps else you can die. I had heard this on radio and TV too.” [Int. 12]*

#### ‘The tipping point’ - deciding to seek help

Symptoms that disrupted daily function, affected quality of life, or was perceived to potentially affect quality of life were important triggers to seek help.

*“I see that the illness keeps getting worse, it is growing bigger, and now my neck is also hurting so bad I just sit till morning comes so I decided to go and see the doctor” [Int. 4].*

*“So when it started paining me and I could not go to the farm, I could not do anything, that is when I went to tell my brother and the same day we went to the doctor” [Int. 1]*

### The health system/facility phase

The time from symptom identification to reporting to KATH ranged from 4 to 24 months.

Six (6) women had their first contact with a health facility less than 3 months after identifying their symptom. The initial contact for all the women was with a non-specialist physician. The referral to KATH was not straightforward for all the women. Some of them saw up to 3 other physicians, including travelling to other towns for diagnostic investigation before presenting to the KATH breast clinic.

The time it took to navigate the course from the point of initial healthcare contact to KATH was closely linked to their economic, family and social roles as women. Some economic and sociocultural activities were prioritised ahead of seeking care for the breast. For example, some deferred hospital appointments in order to work for more money; some prioritised treatment for other illnesses perceived to affect their functionality; and others prioritised taking care of an ill child over their own health.

*“I had heard about breast disease so even though it was not paining me I had to do something about it…..but it was not worrying me and we had entered the Christmas season too so I wanted to work a little more” [Int. 12]*

“*It has been swollen for over a year, I did not do anything about it…. I was seeing the doctor monthly for my hypertension medication” [Int. 6]*

*“The child’s illness was more serious and so I had to take care of him first, I didn’t consider mine as that serious” [Int. 3]*

The availability of money to pay for care and diagnostic investigations also significantly influenced the time taken to navigate the referral pathway.

*“You see, by the time I was discharged about my leg problem, I had no money on me that is why it took me so long” [Int. 5]*

*“By the time we got to the regional hospital, all our money was finished so the doctor asked us to go back home and mobilise some money for the tests.” [Int. 14]*.

“*Well I wanted to come but the money issues, the money issues” [Int. 2]*

Even when money was available, it was prioritised for other economic, social or family use.

*“Recently too I had gone to pay school fees for two of my children so the money I had, if I came,* (it) *was not enough” [Int. 15]*

The women did not seem to have any problems with their health seeking journey. They believed their decisions and the timing of their actions taken was justified; it could not have been any other way. At the time they had decided to seek medical care, the breast symptom was a singular priority, and nothing was considered a hindrance.

*“Well the time I felt the pain I did not delay, I went to the hospital. They gave me a referral on Tuesday, only Wednesday passed and I travelled here on Thursday. I am okay with how things have gone” [Int. 15].*

*“I am pursuing this because of the children, if I die they will become miserable” [Int. 9]*

“W*ell if you want good for yourself you must spend money” [Int. 7]*

*“I was not bothered about coming here because I want my good health back” [Int. 3]*

*“The distance is far but if it will help me then there is no problem” [Int. 12]*

Essentially, while deferring healthcare was related to ability to perform economic, family and social roles, pursing healthcare was also for the same economic, family and social reasons or goals.

## Discussion

### The personal phase – the prioritization cycle

Breast symptoms that interfere with daily activity or function is a significant factor in deciding that breast disease is serious and needs medical attention [18, 39]. As observed in this study, it is not the physical characteristics of a breast symptom, but how it affects a woman’s function that provokes health seeking. This suggests that the breast symptom is not appraised as an isolated entity, but with regard to its effects on different aspects of a woman’s functionality. How the breast symptom interrupts economic, family and social function is an important factor in defining disease severity and the need for urgent medical attention. The mere presence of the breast symptom may not be alarming enough until it is perceived as a threat to, or actually impacts, functionality.

Breast cancer campaign messages in Ghana generally encourage screening, especially self-breast examination for early signs of breast cancer, and where detected, to immediately seek medical care. The general expectation is that appraisal of a breast cancer symptom should culminate in the decision to seek treatment, and this health seeking action is again anticipated to be immediate – without delay. Reasonably, the responsibility to appraise breast symptoms and follow through to seek medical attention cannot be taken away from the individual. This desired health seeking behaviour has been communicated in diverse ways. In spite of this, delayed health seeking persists. However, having knowledge does not necessarily guarantee timely health seeking behaviours. From their exploration of the cultural model of breast cancer among low-income African American women, Barg and Grier [44] attribute this phenomenon to the different cultural beliefs and experiences that exist about breast cancer. Thus, the intended cognitive and affective response expected from general education on breast cancer may be different from the actual meaning generated during a woman’s interpretation of the message [44]. Even where the intended and actual meanings are congruent, Granek and Fergus [45] in their discussion of issues of agency and liminality associated with women’s symptom appraisal and help-seeking behaviour upon discovery of a breast symptom, assert that women who are not ready to present their breast symptoms to a physician remain ‘deliberately ignorant’ of it because they have other areas in their life that needs attention and that are not hindered by the threat of breast cancer, or the early symptoms of breast disease. Whilst this assertion may be true in some cases, the findings from this study suggest that women are not necessarily deliberately ignorant. Rather, they remain aware of their symptoms but make active choices of prioritising other areas in their life needing attention until such time that seeking care for their breast symptoms is the means to achieve those other previously prioritised choices. In other words, when their perceived priorities are latterly threatened by the breast symptom, seeking medical attention to treat the breast symptoms becomes a means to ensure that those previous priorities are maintained or restored. The decision-making process of health seeking is thus part of an ongoing iterative priority setting process, the ultimate goal of which is to maintain important economic, family and or social function.

Acting on the breast symptom is thus one of the other means (e.g. work, child care) to achieving some economic, social or family goal, such that its place on the priority ladder depends on how it is directly related to achieving these goals. There is an awareness of the breast condition, but this ‘joins the queue’ of other important things that also need to be done. As such, for some women, the timing for health seeking is not delayed. *The timing is right*; the time when the symptom is beginning to threaten economic/family/social goals; the time when if the symptom is not dealt with, other priorities cannot be achieved; the time when health seeking for the breast symptom becomes the means by which the other activities can be sustained; the time when in the regular priority setting agenda, it has become the number one activity to be done in the daily pursuit of economic, family and social function.

### The health facility phase

Breast cancer treatment tends to be offered in specialised centres. Referrals are therefore necessary for many women on the health seeking journey. For the women in this study, referrals were not just for treatment but also for investigations to establish diagnosis. This journey translates into time, money and the use of other resources, competing with daily efforts to maintain their economic, family and social functions. When resources are limited, the decision to commit any of these resources to health seeking is further delayed, especially when the symptom is not perceived a threat to function. Already existing narratives about the cost burden and time consuming nature of breast cancer care further worsen this inertia. Even when the health seeking journey has been initiated, activities such as diagnostic investigations may be deferred as economic, family or social goals come up and compete for resources.

Probably, a clear understanding of the false economy of delaying treatment in competition with the very economic, family and social goals they hold so dear could have led to different health seeking choices and this should feature prominently in breast cancer education.

## Conclusions

Deciding to seek care and pursue treatment for breast cancer symptoms may be much more complicated than it appears. Economic, family and social function significantly drive the health seeking process both at the personal and health facility phases of health seeking.

It may be useful to adapt breast cancer campaign messages delivered by national and local public health agencies to incorporate these functional goals and their role in symptom appraisal and decision making, rather than focus on the breast symptom as an isolated entity. A national document on breast cancer education approved by all stakeholders should be developed to serve as the basis for breast cancer campaign messages delivered on all media and local group platforms to ensure uniformity and consistency of the information given to women.

Further, there is need for more research to explore how health workers, non-governmental organizations and other stakeholders who are involved in breast cancer campaigns and treatment can provide targeted communication and counselling support to women to undergo breast cancer investigations and treatment.

### Limitation

There was likely some recall bias as the women were recollecting activities that had occurred several weeks to months prior to arriving at the breast clinic.

Interview transcripts could not be returned to participants for feedback because most of them could not be traced. This could potentially affect rigour. However, immediately after each interview, a quick recap of the interview was always fed back to the interviewee before closing off the interview.

The interviewer’s background as a surgeon who had worked in the breast clinic and with breast cancer patients, and as a researcher with academic interests, may have influenced her qualitative analysis. However, the other authors were at liberty to comment on the qualitative findings.

## Data Availability

We do not have participants’ consent to share their data – including anonymized forms of complete interview recordings and their transcriptions.

## Abbreviations

WHO: World Health Organisation
KATH: Komfo Anokye Teaching Hospital
CHRPE: Committee on Human Research, Publication and Ethics
SBE: Self breast examination
